# Myeloid Dendritic Cells are Potential Players in Human Neurodegenerative Diseases

**DOI:** 10.3389/fimmu.2015.00632

**Published:** 2015-12-16

**Authors:** Paola Bossù, Gianfranco Spalletta, Carlo Caltagirone, Antonio Ciaramella

**Affiliations:** ^1^Department of Clinical and Behavioral Neurology, IRCCS Santa Lucia Foundation, Rome, Italy; ^2^Menninger Department of Psychiatry and Behavioral Sciences, Baylor College of Medicine, Houston, TX, USA; ^3^Department of Neuroscience, University of Rome Tor Vergata, Rome, Italy

**Keywords:** Alzheimer’s disease, Parkinson’s disease, blood-derived myeloid cells, monocyte-derived dendritic cells, dendritic cell precursors, blood DCs

## Abstract

Alzheimer’s diseases (AD) and Parkinson’s diseases (PD) are devastating neurodegenerative disturbances, wherein neuroinflammation is a chronic pathogenic process with high therapeutic potential. Major mediators of AD/PD neuroimmune processes are resident immune cells, but immune cells derived from periphery may also participate and to some extent modify neuroinflammation. Specifically, blood borne myeloid cells emerge as crucial components of AD/PD progression and susceptibility. Among these, dendritic cells (DCs) are key immune orchestrators and players of brain immune surveillance; we candidate them as potential mediators of both AD and PD and as relevant cell model for unraveling myeloid cell role in neurodegeneration. Hence, we recapitulate and discuss emerging data suggesting that blood-derived DCs play a role in experimental and human neurodegenerative diseases. In humans, in particular, DCs are modified by *in vitro* culture with neurodegeneration-associated pathogenic factors and dysregulated in AD patients, while the levels of DC precursors are decreased in AD and PD patients’ blood, possibly as an index of their recruitment to the brain. Overall, we emphasize the need to explore the impact of DCs on neurodegeneration to uncover peripheral immune mechanisms of pathogenic importance, recognize potential biomarkers, and improve therapeutic approaches for neurodegenerative diseases.

## Introduction

Neurodegeneration is a pathologic process leading to the loss of structure and function of neuronal tissue. The two most frequent neurodegenerative disturbances affecting humans are Alzheimer’s (AD) and Parkinson’s disease (PD), respectively, leading to dementia and movement disorders. They are age-dependent and progressive diseases with distinct clinical and pathological features and different types of neurons and brain areas resulting affected, but with some overlapping pathologic mechanisms. Both diseases are typified by the presence in the brain of the peculiar age-related accumulation of specific misfolded molecules, namely amyloid β peptides (Aβ) and tau protein in AD, and α-synuclein protein (α-syn) in PD. Such molecules are widely hypothesized to be primary cause of disease and appear implicated in several key pathogenic mechanisms (1, 2). The presence of abnormal molecular deposits is accompanied by the activation of resident brain immune cells (microglia) and release of pro-inflammatory mediators. Thus, despite both disturbances appear primarily triggered by non-immune causes, inflammation has been increasingly described as a common and central pathogenic factor of the neurodegenerative process (3–5). It is generally accepted that an impaired ability of brain immune cells to clear dangerous substances and damaged tissue promotes the establishment of inflammation, whose persistence in turn may lead to neurodegeneration progression through a self-perpetuating chronic process that ends up with brain tissue damage, but the precise mechanisms underlying the multifaceted role of inflammation in the disease are still intensely debated (6, 7). Recent genetic and expression studies have identified a number of genes connected with inflammation and immune response as risk factors for the development of both AD and PD (8, 9), reinforcing the view that inflammation is a major causative factor and not just a consequence of neurodegeneration. This is especially true in the sporadic forms of AD/PD, which are much more common than the familial forms (10–12). Furthermore, the use of anti-inflammatory drugs may delay or prevent the onset of AD and PD, validating the concept that inflammation is a good therapeutic target (13–15), even though some clinical trials generated disappointing results (16). Such inconsistency is attributed to the incomplete understanding of the interactions occurring between immunity and central nervous system (CNS), including the potential regulative functions of blood-derived immune cells (17). In this regard, the modern concept of the brain’s immune privilege based on a dynamic interaction between peripheral immune system and CNS (18, 19) and the very recent discovery of meningeal lymphatic vessels in the brain (20) validate the idea that the bidirectional communication between CNS and systemic immunity is more resourceful than before considered. Although no massive infiltration of blood-derived immune cells is reported in neurodegeneration, it is growing the thought that the recruitment of bone marrow-derived immunocompetent cells from the systemic circulation to the brain is an important event, which could regulate neuroinflammatory response and influence neurodegeneration (21).

Given such premises, dendritic cells (DCs), acting as key controllers of the immune response and inflammation, may be crucially involved in maintaining brain immune surveillance, helping resident microglia in response to insults, and regulating the local and systemic immune response during neuroinflammation (22). Here, we will examine the current evidence that converges to propose DCs as potential disease-relevant cell types in both AD and PD.

## Peripherally Derived Immune Cells in Neurodegeneration

Microglia, the brain-resident mononuclear phagocytic cells, play a central role in AD and PD chronic inflammation, with insights in disease susceptibility, pathophysiology, diagnosis, prognosis, and therapy (23). Although normally microglia have a protective role linked to their ability to clear harmful material, their activation in AD and PD has been related to the brain accumulation of abnormally aggregated proteins, increased production of inflammatory factors, and neuronal loss. More recent data suggest that during neurodegeneration, microglia acquire a dysfunctional rather than activated phenotype, characterized by defective clearance and loss of neuronal support capability. Although microglia are main drivers of neurodegenerative disease, their precise role is still a matter of debate and, importantly, it is now emerging that they may not be the only effectors of brain immune response. Sustained by the revised concept of the immunoprivileged brain, a contribution to neurodegeneration of additional mediators of the innate immune system beside microglia is widely hypothesized (17, 24), and systemic inflammation is considered an exacerbating force in neurodegenerative diseases (25, 26).

The notions that aging and neuroinflammation promote disturbance of the blood–brain barrier (BBB) and that neurovascular dysfunctions lead to alteration of the crosstalk between different cell types at the brain–blood interface in both AD and PD (27, 28) make conceivable the participation of peripheral cells in neurodegeneration.

Myeloid cells that can mediate the brain innate immune response beside microglia include perivascular cells, meningeal macrophages and blood-borne monocytes, macrophages, and DCs. All these cell types are not homogeneous populations; they differ in terms of origin, function, and fate, respond in a variable way to environmental changes, and act in concert during neurodegeneration (29). While the characteristics of the different myeloid cell populations participating in brain homeostasis and inflammation have been reported elsewhere (30), here we describe some of the most recent evidence sustaining the value of blood-derived monocytic cells in AD and PD.

Many of the genes recently identified to be associated with both familial and sporadic forms of AD and PD have been found to be also expressed and play important roles in peripheral innate immune cells. Specifically, *CD33* and *TREM2* gene variants have been reported as significant risk factors for AD (31, 32) and associated also with PD (33). These genes are strictly coupled with myeloid cell functions leading to the clearance of misfolded molecules, and are expressed, other than on microglia, also on peripheral myeloid cells. Consistently, a gene expression profile study shows overexpression of AD and PD risk alleles specifically in patients’ blood monocytes (34).

Several studies report that monocytic cells, able to infiltrate the degenerated brain following specific signaling pathways, may be capable to modulate AD progression (35). Consistently, in real-time *in vivo* imaging studies, vascular Aβ clearance is carried out by patrolling monocytes (36), according to previous results showing that peripherally derived myeloid cells are protective in AD through mechanisms of brain recruitment involving MCP-1 chemotaxis (37–39). At variance, peripherally derived macrophages have a detrimental role in *TREM2*-deficient AD mice (40), suggesting that the function of infiltrating myeloid cells may be complex and dependent on cell subset and inflammatory context considered. Clinical evidence on the involvement of peripherally derived mononuclear phagocytes comes from the observations that AD macrophages infiltrate the brain and damage the BBB, and in the blood they show abnormal cytokines release, increased apoptosis, and impaired ability to engulf Aβ (41, 42). Regarding PD, monocyte infiltration is involved in aggravation of neurodegeneration in murine transgenic models (43). Similarly, circulating blood monocytes have been found altered in PD patients as for transcriptome profile and function, and their hyperactivity has been shown to correlate with disease severity (44). Consistently with AD, the CCL2–CCR2 system appears to play a dominant role also in monocyte recruitment to PD patients’ brain (45). However, the precise identity and dynamic of infiltrating myeloid immune cell subpopulations as well as their role in the disease context is to date still vague. In fact, despite novel molecular biological tools have shed some light on the origin and functions of microglia versus other myeloid cell populations in the CNS (46), the identification of infiltrating myeloid subsets (including DCs) is hard in neurodegeneration, especially in humans, where restricted cell numbers, high morphological and functional heterogeneity linked to environmental changes, and lack of specific markers (47) prevent to make accurate distinctions.

## Evidence for Dendritic Cell Participation in Neurodegeneration

Dendritic cells are chief orchestrators of the immune response and main link between innate and adaptive immune response (48). They form a heterogeneous cell population, located in different body tissues, with diverse immunological functions. Considering the main properties of DCs, such as migratory abilities, pivotal role in looking out potentially harmful factors, and ability to regulate both innate and acquired immunity and to resolve responses potentially harmful if left uncontrolled, they may be key players in brain immune surveillance and good candidates as participants in neuroinflammation and neurodegeneration. Linked to their key activity to educate T cells in driving the immune response, DCs may stimulate lymphocytes during their entry into brain and activate an adaptive immune response during AD and PD pathogenic processes. Indeed, dysregulation of the adaptive immune response, in terms of abnormal brain T cell activation and infiltration, has been reported in both experimental and human conditions of AD and PD (49–53).

### Brain DCs

Regarding the identification of DCs in the brain, as exhaustively reviewed by Colton (54), a number of studies mainly performed in animals have provided evidence for their presence within CNS. Generally defined as brain DCs, these cells have dendritic morphology and express major histocompatibility complex class II molecules (MHCII) and leukocyte integrin CD11c, are heterogeneous and may exert complex immunological functions, and likely derive from circulating DC precursors, rather than from within the brain, as suggested by their juxtavascular location in the meninges and the choroid plexus (55). Brain DCs are present in CNS compartments, including cerebral spinal fluid (CSF), meninges, choroid plexus, and perivascular spaces of both rodents and humans (56–58), where they may act as immune sentinels at the interfaces between the brain and periphery. Although low in the steady state, DCs’ frequency increases in CNS under neuroinflammatory conditions (57, 59, 60) and during aging (61), raising the possibility of their participation in neurodegenerative processes.

Though the DC presence in AD or PD brain parenchyma has not definitively proved so far, it has been hypothesized in murine models of AD (62), demonstrated in aged yellow fluorescent protein transgenic mice (61) and only guessable in postmortem human AD brain tissue, where in glial cells, a phenotypic change, recalling that of activated DCs, was observed (63).

While protective activities linked to their ability to clear Aβ have been suggested for CD11c^+^ cells in AD mouse models (64, 65), indirect data suggest that DCs are pivotal in PD pathogenesis, where an immune response to self-antigens may be a causative factor (66, 67).

Additional insights about DC participation in human neurodegeneration come from *in vitro* experiments addressed to explore how Aβ peptides impact DC function in humans, as well as from *in vivo* analysis of DC precursors’ frequency in blood of patients with neurodegeneration (Table 1).

**Table 1:** ***In vitro* and *in vivo* evidence for DC participation in human neurodegenerative diseases**.

Disease	DC type	Model	Analysis	Main finding	Reference
AD	Monocyte-derived	*In vitro* triggering with Aβ_25–35_	Phenotype, cytokine production, T cell activation	↓ MHCII	Schmitt et al. (68)
	Monocyte-derived	*In vitro* generation with Aβ_1–42_ (long lasting stimulation)	Phenotype, phagocytosis, cytokine production, T cell activation	↑ Cell recovery; ↑ antigen uptake; ↑ IL-1β/IL-6/IL-18; ↓ IL-12/IL-10; ↓ MHCII; ↓ APC ability	Ciaramella et al. (69)
	Monocyte-derived	*In vitro* generation from AD patients’ monocytes	Phenotype, phagocytosis, cytokine production, T cell activation	↑ Cell recovery; ↑ ICAM-1; ↑ IL-6; ↓CD40; ↓ APC ability	Ciaramella et al. (70)
	Monocyte-derived	*In vitro* generation from AD patients’ monocytes triggering with Aβ_1–42_	Phenotype, phagocytosis, cytokine/neurotrophin production, T cell activation	↓ BDNF	Ciaramella et al. (71)
	Blood precursors	*Ex vivo* precursors from AD patients’ blood	Flow cytometry in blood cells (Lin 1^-^/MHCII^+^/CD11c^+^ or CD123^+^)	↓ mDCs; association with AD symptom severity	Ciaramella et al. (2015, submitted)
PD	Blood precursors	*Ex vivo* precursors from PD patients’ blood	Flow cytometry in blood cells (Lin 1^-^/MHCII^+^/CD11c^+^ or CD123^+^)	↓ mDCs; ↓ pDCs; association with PD symptom severity	Ciaramella et al. (72)

### *In vitro* Monocyte-Derived DCs

As renowned cell model to analyze human DC functional response (73), *in vitro-*generated monocyte-derived DCs (moDCs) have been used under neurodegenerative specific conditions. MoDCs triggered with Aβ_25–35_ peptide show only a slight decrease in surface expression of MHCII (68). At variance, when the same cells are differentiated in the presence of Aβ_1–42_ in a long-lasting stimulation state, which presumably better mimics AD conditions, Aβ induces increase in cell survival and soluble antigen uptake, elevated production of IL-1β, IL-6, and IL-18, but a decrease in MHC expression and ability to activate T cells (69). Interestingly, moDCs obtained from AD patients, as compared to age-matched control subjects, show more pronounced pro-inflammatory features and reduced antigen-presenting ability (70), and notably they produce lower levels of BDNF following Aβ_1–42_ stimulation (71). All together, these findings, other than advocate for the suitability of this cell model to investigate the mechanisms of myeloid involvement in neurodegeneration, also indicate that under AD conditions, DCs might contribute to brain damage by mechanisms of overactivation of inflammatory responses and Aβ-mediated reduction of trophic support to neurons, suggesting that they may be mediators of AD neurodegeneration.

### Blood DCs

To exercise their ability to survey the brain and activate adaptive immune response, precursors of DCs should migrate from the bone marrow to CNS sites. In humans, the most suitable populations of circulating cells with such features are the two main subpopulations of blood immature DCs, namely myeloid (mDCs; lin^−^ CD11c^+^ MHCII^hi^, CD123^lo^) and plasmacytoid (pDCs; lin^−^ CD11c^−^ MHCII^mod^, CD123^hi^) cells, similar to those described in the CSF (57, 74, 75). Thus, to confirm the potential relevance of DCs in neurodegenerative diseases, frequency and phenotypic abnormalities of the two blood DC precursor subsets, mDCs and pDCs, and their relationships with disease outcome have been evaluated in both AD and PD patients. Preliminary results from our research group indicate a significant drop of myeloid DC precursors in blood of AD patients, occurring in association with increased severity of disease symptoms (Ciaramella et al. 2015, submitted). Similarly, blood DC levels, regarding both mDC and pDC subsets, are decreased in PD patients in association with increased impairment of motor functions, suggesting innovative exploitations of DC monitoring as a clinically significant tool for neurodegenerative diseases (72). Although the reduction of blood DCs may be due to different circumstances, including alterations in either viability, mobilization, or differentiation of DCs from their progenitors, it may likely depend on their recruitment from circulation to degenerating brain, similarly to what observed also in other brain diseases.

**Figure 1:**
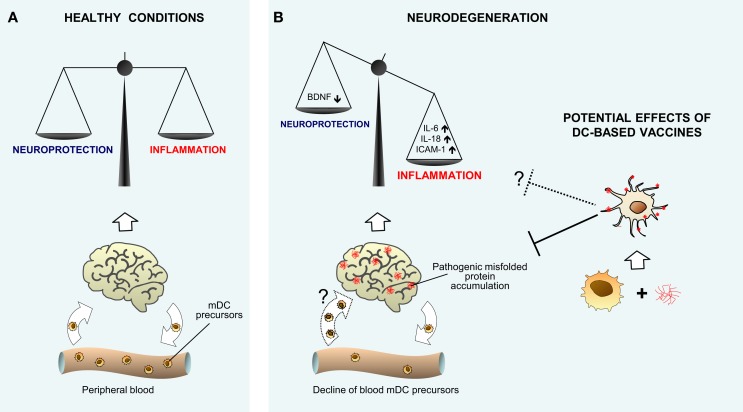
**Hypothetical model showing the role of myeloid DCs in neurodegenerative diseases. The cartoon recapitulates our view of the potential role of myeloid DCs in neurodegenerative diseases, as both possible participants in promoting inflammatory neuroimmune processes, and supposed tools to perform vaccine therapy**. **(A)** Given their nature as key controllers of the immune response and inflammation, mDCs may participate in maintaining brain immune surveillance and controlling the delicate homeostatic balance between protective and inflammatory neuroimmune processes in normal healthy conditions. **(B)** As suggested by human *in vitro* data, during neurodegeneration, mDCs may be involved in promoting imbalance between protective and inflammatory neuroimmune processes. The decline of DC precursors observed *in vivo* in peripheral blood of patients with neurodegenerative diseases may be a consequence of cell recruitment to the diseased brain (illustrated as a dotted arrow), where DCs may acquire a dysregulated phenotype and contribute to the inflammatory milieu. The potential therapeutic use of DC vaccination in neurodegeneration is depicted [right hand side of **(B)**] on the basis of animal models’ results. Myeloid DCs differentiated and expanded from peripheral precursors, and specifically targeted *in vitro* against misfolded proteins, may trigger an immune response that promotes the clearance of brain aggregates and attenuates symptoms, possibly even restoring the neuroinflammatory homeostasis (illustrated as a dotted line).

Collectively, the above described *in vitro* and *in vivo* human studies lead to speculate that in AD and PD, a percentage of blood DCs moving from peripheral blood may reach the brain of patients, probably at choroid plexus or meninges level, where they may sample CSF content for brain antigens (including Aβ), acquire a dysregulated phenotype, and contribute to the inflammatory milieu, playing a putative pathological role in neurodegeneration, as depicted in Figure 1.

Additional studies should be addressed to better understand whether, after reaching the brain, DCs may function to locally regulate infiltrating activated T cells or carry antigens to CNS-draining cervical lymph nodes. Furthermore, although an active research is addressed to specifically understand how adaptive immune response participates in this scenario (76), details about DC role in orchestrating the induction of immunity and tolerance in neurodegenerative diseases are so far unknown.

## Potential Therapeutic Uses of Dendritic Cells in Neurodegeneration

Given the potential key role of DCs in regulating brain immune response during neurodegeneration, these cells should be considered as potentially useful target for therapeutic approach of neurodegenerative diseases. Nearly all studies in this direction regard the use of DCs as tools for vaccination purposes, rather than targets to modify the immune responses, since the latter issue is still far from being resolved. Several optimized immunization approaches addressed to eliminate accumulation of misfolded proteins have been considered in the last years as a promising therapeutic strategy for neurodegenerative diseases. DCs may be good candidates as potent antigen-presenting cells appropriately loaded *in vitro* with suitable peptides of misfolded proteins, and made able to preferentially elicit a non-inflammatory-specific immune response (77). Thus, in AD animals, Aβ-sensitized DCs have been used to reduce Aβ accumulation and attenuate cognitive deficits (78), and to prevent AD pathology when used in combination with other cells to reverse immunosenescence (79). A recent study addressed to investigate the molecular mechanisms allowing T cells to specifically target Aβ-loaded brain areas following Aβ immunization of AD mice proposes that DCs play a role in regulating Aβ-specific T-cell entry into the brain at leptomeningeal and perivascular spaces (80). Similarly to the AD state, the removal of α-Syn has been assumed to have the potential to modify the course of PD, and α-Syn-stimulated DCs injected into PD transgenic mice induce the production of anti-α-Syn antibodies and improve the animal locomotor functions (81). The proposed therapeutic use of DCs, as suggested by animal studies, is illustrated in Figure 1B (right hand side). However, in order to translate into clinic, further studies are needed to verify in humans the potential ability of this approach to both provide an effective adaptive immune response against misfolded proteins and counteract the putative pathological role of endogenously dysregulated DCs.

## Concluding Remarks

Within an evolving picture of the immune-to-brain crosstalk, the systemic immune response appears integral to the function of brain-resident immune cells during neurodegeneration, with myeloid immune cells holding a pivotal role. Among those, blood-derived DCs may participate, though their involvement in neurodegeneration awaits experimental verification, especially in humans. The advent of new potent technological tools, including microarray technology, next generation sequencing transcriptome, and epigenetic analysis, may help in identifying the function of DCs in the initiation and/or regulation of the brain-specific immune response.

In conclusion, answering the questions about DC infiltration in AD/PD brain and DC function in human disease progression might be essential for neurologists to better understand the neurodegeneration pathophysiology, develop biomarkers, and improve therapeutic approaches for the most common and devastating neurodegenerative diseases of the modern society.

## Author Contributions

PB, GS, CC, and AC provided substantial contribution to the conception and design of the manuscript. PB drafted the manuscript and PB, GS, CC, and AC revise it critically for important intellectual content. PB, GS, CC, and AC provided the final approval of the version should be published. PB, GS, CC, and AC agreed to be accountable for all aspects of the work in ensuring that questions related to the accuracy or integrity of any part of the work are appropriately investigated and resolved.

## Conflict of Interest Statement

The authors declare that the research was conducted in the absence of any commercial or financial relationships that could be construed as a potential conflict of interest.
